# Preconception diet quality and modified natural cycle in vitro fertilisation outcomes

**DOI:** 10.1017/jns.2024.97

**Published:** 2025-01-23

**Authors:** Janine P.M. Faessen, Irene Homminga, Marion E.C. Buso, Ruxandra A. Nagy, Jannie van Echten-Arends, Edith J.M. Feskens, Uwe J.F. Tietge, Annemieke Hoek, Elske M. Brouwer-Brolsma

**Affiliations:** 1 Division of Human Nutrition and Health, Wageningen University & Research, Wageningen, The Netherlands; 2 Department of Obstetrics and Gynaecology, University of Groningen, University Medical Centre Groningen, Groningen, The Netherlands; 3 Division of Clinical Chemistry, Department of Laboratory Medicine, Karolinska Institutet, Stockholm, Sweden; 4 Clinical Chemistry, Karolinska University Laboratory, Karolinska University Hospital, Stockholm, Sweden

**Keywords:** Diet quality, Preconception, Modified natural cycle in vitro fertilisation, Infertility, Pregnancy, IVF, in vitro fertilization, MNC-IVF, modified natural cycle in vitro fertilization, UMCG, University Medical Centre Groningen, DHD2015, Dutch Healthy Diet 2015, BMI, Body Mass Index, ART, assisted reproduction technologies, ICSI, intra-cytoplasmatic sperm injection

## Abstract

Lifestyle has been associated with in vitro fertilisation (IVF) success rates, but studies on diet and IVF outcomes are inconclusive. We studied associations between adherence to the Dutch guidelines for a Healthy diet 2015 and pregnancy chances among women receiving modified natural cycle in vitro fertilisation (MNC-IVF). This prospective cohort study utilised data from 109 women undergoing MNC-IVF between 2014 and 2018 at University Medical Centre Groningen enrolled in a study examining associations between metabolic profile of follicular fluid and oocyte quality. Adherence to dietary guidelines was assessed by daily food records quantified based on the Dutch Healthy Diet (DHD) 2015 Index. IVF outcomes (i.e. positive pregnancy test, ongoing pregnancy, and live birth) were obtained from patient records. Statistical analyses involved Cox proportional hazard regression analyses while adjusting for maternal covariates age, smoking, and Body Mass Index (BMI), and stratified for treatment, age, BMI, and energy intake. Women were 31.5 ± 3.3 years old, and had a BMI of 23.5 ± 3.5 kg/m^2^. Higher DHD2015 adherence was linked to a reduced probability of achieving an ongoing pregnancy (HR = 0.77, 95%CI: 0.62–0.96), live birth (HR = 0.78, 95%CI: 0.62–0.98), and showed a non-significant trend towards a lower probability of a positive pregnancy test (HR = 0.85, 95%CI: 0.71–1.01). Associations were particularly present among women undergoing MNC-ICSI (*n* = 87, p-for-interaction = 0.06), with shorter duration of infertility (*n* = 44, p-for-interaction=0.06), being overweight (*n* = 31, p-for interaction = 0.11), and having higher energy intakes (*n* = 55, p-for-interaction = 0.14). This explorative study suggests inverse trends between DHD2015 adherence and MNC-IVF outcomes, encouraging well-powered stratified analyses in larger studies to further explore these unexpected findings.

## Introduction

Infertility affects up to 15% of couples trying to conceive and a significant proportion of these couples eventually need assisted reproduction technologies (ART) to achieve a pregnancy.^(1)^ In-vitro fertilisation (IVF) and intra-cytoplasmatic sperm injection (ICSI) are among the most performed ART methods to achieve a live birth.^(1,2)^ Success rates of ART are relatively low (i.e. live birth rates of 20%–25% per started cycle) and the mental, physical, and financial impact of these procedures is high.^(3,4)^ Taking these challenges into consideration,^(2–4)^ a better understanding of potentially modifiable factors associated with natural and ART pregnancy rates is needed.

Recent literature relates unexplained infertility in women to preconception lifestyle factors^(5)^ such as Body Mass Index (BMI),^(6)^ smoking,^(7)^ physical activity,^(8)^ and dietary habits.^(9)^ Several observational studies suggest positive associations between the consumption of specific food groups or nutrients and maternal fertility,^(10–12)^ particularly for the consumption of whole grains,^(12)^ polyunsaturated fatty acids,^(11)^ protein,^(13)^ dairy,^(14)^ and supplemental intake of iron and folic acid.^(10)^ In contrast, trans-unsaturated fat intake,^(15)^ red and processed meat consumption, and low fruit and vegetable intake have been suggested to increase the risk of maternal infertility.^(7)^ Associations between dietary patterns and infertility have been examined less extensively.^(16–20)^ Exploring dietary patterns helps to account for possible relevant complex interactions between foods and nutrients, such as synergic, antagonistic and cumulative effects,^(21,22)^ which may even better predict pregnancy outcomes than individual dietary components.^(23)^

Various studies explored potential associations between dietary patterns and pregnancy outcomes.^(16–20)^ A systematic review from 2022 including 9 observational studies on the association between dietary patterns and IVF outcomes indicated associations between adherence to the MedDiet, a Dutch ‘preconception’ diet and a ‘pro-fertility’ diet, and biochemical pregnancy, clinical pregnancy, and live birth.^(16)^ However, due to large heterogeneity between studies as well as suboptimal adjustment for confounders, no strong conclusions could be drawn.^(17–20,24)^ Moreover, all of the included ART studies focused on conventional IVF with hyperstimulation procedures.

An alternative to conventional IVF is modified natural cycle (MNC)-IVF.^(25)^ MNC-IVF follows a more natural approach that only requires a fraction of the hormones needed for conventional IVF. Using MNC-IVF as a model has the advantage that the sequence of single follicle development, one oocyte at follicle punction, single embryo development, and reproductive outcomes, such as ongoing pregnancy rates and live birth rates, resemble a more natural ovulatory cycle as opposed to multiple follicles in conventional IVF. Although still a form of assisted reproduction, MNC-IVF offers a valuable model to study diet’s potential impact under conditions that more closely mimic natural conception. Therefore, this study aims to explore the association between diet quality, assessed using the Dutch Healthy Diet (DHD)-2015 index,^(26)^ and pregnancy outcomes in women undergoing MNC-IVF.

## Materials and methods

### Participants and study design

This study was conducted using data from a Dutch prospective observational study, registered under NL47569.042.13, which aimed to investigate the relevance of biomarkers and nutritional patterns in the pre-ovulatory period for reproductive outcomes.^(27)^ Participants were recruited between October 2014 and March 2018 by their gynaecologist or IVF physician, with recruitment occurring after the decision to start MNC-IVF treatment, ensuring no treatment delays. Couples were eligible for MNC-IVF if they were 35 years or younger, started their first IVF/ICSI treatment ever or after a previous pregnancy (either spontaneous or through ART), and had normal menstrual cycles (a self-reported regular cycle of 26 up to 35 days). Up to six MNC-IVF study cycles were conducted over consecutive months. However, some couples had breaks in between study cycles and thereby the maximum treatment period in this study was 22 months. Women with polycystic ovary syndrome (PCOS) or applying for egg cell donation were not eligible. During their initial visit to our department, each couple completed a baseline questionnaire as part of standard procedures. This questionnaire covered various aspects including medical history, medication use, smoking habits (non-smoker/previous smoker/current smoker), alcohol intake, duration of infertility, and whether the infertility was primary or secondary.

### Population for analysis

**Fig. 1.**
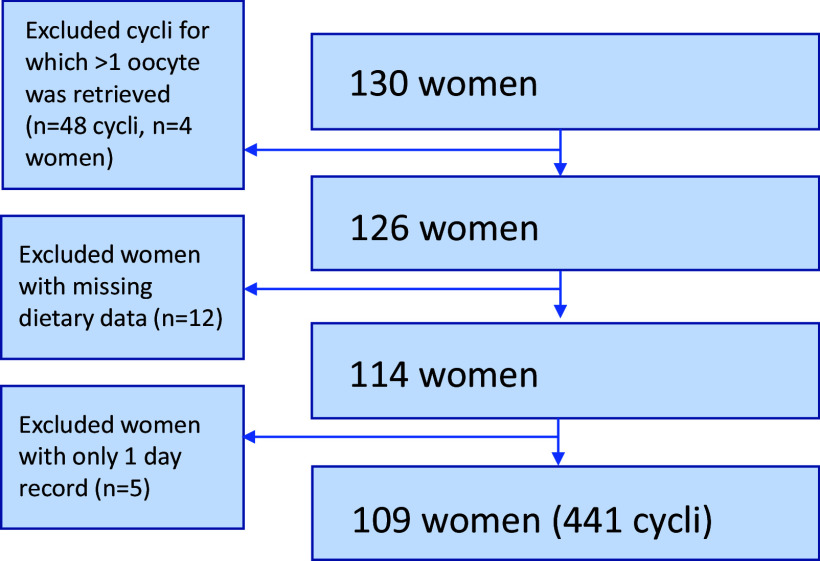
Population for analysis.

As IVF cycles resulting in multiple follicles could lead to more oocytes at retrieval and hence higher pregnancy chances, these study cycles were excluded from the analyses (*n* = 4).^(28)^ Women with missing dietary data (*n* = 12) as well as women with a single-day food record were excluded (*n* = 5). To identify potentially inaccurate dietary intake data, we followed the cut-off points recommended by Willett and colleagues. This process included checking whether the average daily energy intake per woman, calculated by averaging all food records provided by each individual woman, was below 500 kcal or exceeded 3500 kcal.^(29)^ For the current data analyses, study cycles for which both IVF, as well as dietary data, were available were included, resulting in data from 109 women with a total of 441 MNC-IVF study cycles (Figure 1).

### Dietary assessment

Participants recorded their food intake using the Eetmeter mobile app from the Dutch Nutrition Centre^(30)^ for approximately four days, starting from the day a dominant follicle of at least 14 mm was detected until the day before ovum pick-up during their first treatment cycle. The exact number of food records completed by a woman depended on the timing of achieving optimal follicle growth, which ranged between 2 and 13 days. Due to limited Eetmeter output, all dietary data was manually transferred to the web-based application ‘Compl-eat’^(31)^ using the Dutch food composition table (NEVO) codes and portion sizes provided by the Dutch Nutrition Centre. Average daily intakes were calculated by multiplying food intake levels by their nutrient composition according to the NEVO 2016,^(32)^ and foods were combined in food groups based on the classification used in this table^(32)^. Furthermore, individual food items were categorised into food groups for calculation of the DHD2015-score.^(26)^ Some intakes were recorded as recipes or combined dishes, and combined dishes were translated into individual ingredients based on the best-matching available recipe. Food records were checked on plausibility by the research team in consultation with research dieticians.

### Dutch healthy diet index 2015 (DHD2015-index)

The DHD2015-index is an index for calculating the diet quality of healthy adult inhabitants of the Netherlands based on the Dutch Guidelines for a Healthy Diet 2015 for adults.^(26,33)^ The DHD2015-index consists of 15 food-based components for which an individual can score 0 to 10 points per component; a higher score indicates higher diet quality.^(26)^ Adequacy components of the index were scored high for high intake levels (e.g. fruit, legumes, and fish) while the moderation components (e.g. processed meat, sweetened beverages, and fruit juices) scored low for high intake levels. Ratio components reflect the replacement of a less desired food with healthier options (e.g. liquid instead of solid fats).^(26)^ Our food records lacked essential details on sodium (added table salt) and coffee intake (filtered or unfiltered), resulting in their exclusion from the index used for analyses. Consequently, the maximum of each DHD2015 component combined was 130 points (Table 1). Each woman was assigned a personal diet quality score, calculated as the sum of the DHD2015-score of each component at cycle 1 of MNC-IVF/ICSI treatment. For reference, previous studies performed in the Netherlands reported DHD scores of 80.7 ± 1.6 out of 130^(34)^ and 79.4 ± 16.0 out of 150.^(26,35)^

**Table 1.** Overview of DHD2015-index components

	Component	Minimum score 0	Maximum score 10
1	Vegetables	0 g/d	≥200 g/d
2	Fruit	0 g/d	≥200 g/d
3	Grains*	No whole grains OR ratio whole grains to refined grains ≤0.7	No refined grains OR ratio whole grains to refined grains ≥11
4	Legumes	0 g/d	≥10 g/d
5	Nuts	0 g/d	≥15 g/d
6	Dairy**	0 g/d OR ≥750 g/day (5 portions)	300 – 450 g/d
7	Fish***	0 g/d	≥15 g/d
8	Fats and oils	No consumption of liquid fats OR ratio of liquid fats to solid fats ≤0.6	No consumption of solid fats OR ratio of liquid fats to solid fats ≥13
9	Red meat	≥100 g/d	≤45 g/d
10	Processed meat	≥50 g/d	0 g/d
11	Sugar-sweetened beverages and fruit juices	≥250 g/d	0 g/d
12	Alcohol	>20 g/d	<10 g/d
13	Tea (green or black)	0 g/d	≥450 g/d

* For this component 5 points could be acquired from a. whole grain consumption and 5 points from b. ratio of whole grain products and refined products.

** Maximum of 40 g cheese could be included.

*** Maximum of 4 gram lean fish could be included.

### MNC-IVF

MNC-IVF was performed at the University Medical Centre Groningen (UMCG) as previously described by Pelinck et al.^(36)^ In brief, the natural menstrual cycle was monitored by transvaginal ultrasound starting at cycle day 8. Once a dominant follicle of ≥ 14mm diameter was detected by transvaginal ultrasound, the patient started daily hormone injections to suppress premature ovulation (**gonadotropin-releasing hormone (GnRH) antagonist** (Cetrotide^R^), 0,25mg/day) and to support follicular growth (recombinant gonadotropin alfa, 150 IU/day). Ovum pick-up was scheduled within 1–2 days after detecting a dominant follicle of ≥18 mm. This occurred within 2 days if endogenous LH (luteinizing hormone) levels were < 30 IU/l, and within 1 day when levels were >30 IU/l, as measured in peripheral blood. Triggering was achieved using 10,000 IU hCG (PregnylR, Organon, The Netherlands). Oocyte pick-up occurred 34 hours post-hCG triggering. Subsequently, the oocyte was inseminated either by incubation with culture medium containing spermatozoa (IVF) or by intracytoplasmic sperm injection ((ICSI), in case of male infertility.^(37)^ If fertilisation and in vitro embryo development were successful, embryo transfer (in the uterine cavity with a catheter) was scheduled two days after ovum pick-up.^(7)^ Luteal support was added in the form of hCG injections of 1500 IU (Pregnyl^R,^ Organon, The Netherlands) on the 5^th^, 8^th^ and 11^th^ day after ovum pick-up. Patients conducted a self-administered pregnancy test at home, using their first-morning urine, seventeen days after ovum pick-up, and reported the results to our department.

### Fertility outcome measurements

Fertility outcomes were assessed during normal MNC-IVF check-ups, including a positive pregnancy test two weeks after embryo transfer (yes/no), ongoing pregnancy with a foetal heartbeat at 7-8 weeks after embryo transfer (yes/no), and livebirth (yes/no).

### Covariates assessment

Participant characteristics were obtained by means of a general questionnaire before the start of treatment and during regular IVF check-ups, including assessment of weight and length, age, smoking habits, cause of infertility, duration of infertility, and received method of treatment. BMI was calculated based on (self-reported) weight in kilograms and length in centimetres (weight/height^2^). Age was recorded based on the first cycle of treatment. Smoking habits were categorised into no smoker and current or past smoker based on answers to a questionnaire at the intake visit. The cause of infertility was subdivided into male factor, tubal factor and unexplained infertility. Duration of infertility was defined as the time in months in which the couple has (unsuccessfully) been trying to conceive up until receiving the first cycle of treatment. Method of treatment was either MNC-IVF or MNC-ICSI.

### Statistical analysis

Descriptive characteristics are presented for the total population as well as by DHD2015-index tertile as mean with standard deviations (SD) for continuous variables or median with interquartile range (IQR) if skewed, or as frequencies with percentages (*n*, %) for categorical variables. Differences in descriptive characteristics across tertiles were tested using ANOVA for normally distributed variables, Kruskal–Wallis tests for skewed data and Chi-square for categorical data. We applied Cox proportional hazards models to examine the relationship between the DHD-2015-index and positive fertility outcomes, yielding hazard ratios (HR) and 95% confidence intervals (95% CI) across 1 to 6 study cycles for the women. Survival time was calculated based on the number of study cycles. Potential confounders were based on previous literature on IVF treatments,^(7,9,10,28,38)^ and models were adjusted for age, BMI and smoking of the woman. As BMI and energy intake may be considered proxy variables for diet quality,^(39–43)^ additional Cox proportional hazard analyses were performed for the association between BMI and energy intake, and pregnancy outcomes to substantiate the internal validity of the results. Furthermore, effect modification was investigated for age (low/high based on median age), treatment fertilisation method (MNC-ICSI/IVF), duration of infertility (short/long based on median duration), BMI (normal/overweight) and energy intake (low/high based on median intake) to assess potential different associations between diet and outcomes dependent on these factors.^(16,38)^ P-values for interaction for these potential effect modifiers were assessed by including an interaction term in the adjusted model. Only variables that show (potential) interaction based on the p-value for interaction term, were used as stratifying variables (p < 0.15). P-values were based on two-sided tests and significance was assumed below 0.05. Data were analysed using RStudio (version 2022.12.0).

### Ethical approval

The study participants gave consent for the analysis of their food records, follicle fluid, serum, and MNC-IVF/ICSI outcome measurements. The study was registered in 2014 under the registry number NTR4409, performed at the Reproductive Medicine Unit of the University Medical Center Groningen, and conducted in agreement with the Declaration of Helsinki. The clinical study obtained ethical approval from the local Medical Ethics Committee (METC 2014/007, number NL47569.042.13).

## Results

### Patient baseline demographics and IVF outcomes

Our study population included 109 women with a mean (±SD) age of 31.5 years (±3.3) and BMI of 23.5 kg/m^2^ (±3.5) (Table 2). Forty-five women (41.7%) smoked or were past smokers and 62 (57.9%) consumed alcoholic beverages before treatment. Four (3.7%) women completed 2 food records, 21 (19.3%) completed 3, 52 (47.7%) completed 4 food records, and 32 (29.4%) women completed 5 or more food records. Women had a median diet quality score of 61 (25th–75th percentile: 50–72) out of 130, ranging from a minimum of 19 to a maximum of 103. At baseline, 88% of the couples faced infertility for over 12 months, lasting on average 39 (±24) months. Couples receiving ICSI also had a shorter duration of infertility (35 ± 22 vs. 52 ± 27 months, p < 0.01) (not shown in tables). Male factor infertility was the leading cause (*n* = 75, 69.4%), resulting in the majority of couples opting for ICSI (*n* = 87, 79.8%) undergoing a median of 4 study cycles (range: 2–6). Eventually, 44 out of 109 (40.4%) women had a positive pregnancy test at day 15 after embryo transfer, 33 (30.3%) had an ongoing pregnancy at 7-8 weeks of gestation, and 30 women (27.5%) had a live birth. Women with a higher DHD2015 score (scores equal to or higher than 66) were, on average, 2 years older than women with a low score (scores of 54 or lower). Furthermore, women in the highest tertile had a higher number of study cycles (median 5 vs 4 and 3, respectively). As expected most DHD-2015 sub-scores differed significantly across the tertiles, apart from the scores of dairy, red meat, processed meat and alcohol (Table 2). Higher diet quality scores were reflected by a higher intake of protein, fat, fibre and water, and a lower intake of carbohydrates and especially mono- and disaccharides (Supplemental Table 1). Median DHD-index was not significantly different between women achieving pregnancy and women who did not become pregnant (62 (50–75) vs. 57 (47–66)) (Supplementary Table 2). Women achieving a pregnancy consumed more refined grains (125 (83–200) vs 112 (67–137)) and more sugar-sweetened beverages and fruit juices (246 (113–467) vs 119 (50–314)) compared to women that did not achieve a pregnancy.

**Table 2.** Descriptive overview of total study population and across tertiles of DHD2015-index

	Total	Low DHD2015 (≤54)	Moderate DHD2015 (54-66)	High DHD2015 (≥66)	P-value
Participants (n)	109	36	36	37	
**General characteristics**					
Age, mean (SD)	31.5 (3.3)	30.3 (3.6)	31.9 (3.1)	32.3 (2.9)	0.02
BMI, mean (SD)	23.5 (3.5)	23.8 (3.3)	22.9 (3.1)	23.8 (3.8)	0.50
Current or past smoker before treatment, *n* (%)	45 (41.7)	16 (45.7)	14 (38.9)	15 (40.5)	0.83
Alcohol, *n* (%)	62 (57.9)	19 (52.8)	18 (52.9)	25 (67.6)	0.34
Food Supplements (other than folic acid)#	96 (95.0)	30 (90.9)	34 (100.0)	32 (94.1)	0.37
**Cause of infertility,** ***n*** **(%)**					0.54
Male factor	75 (69.4)	27 (75.0)	24 (68.6)	24 (64.9)	
Tubal (female)	15 (13.9)	5 (13.9)	3 (8.6)	7 (18.9)	
Unexplained	18 (16.7)	4 (11.1)	8 (22.9)	6 (16.2)	
Method, IVF (first known method), n (%)	22 (20.2)	7 (19.4)	6 (16.7)	9 (24.3)	0.71
Duration infertility, months mean (SD)	39 (24)	37 (23)	41 (26)	37 (24)	0.56
Number of study cycles, median (IQR)	4 (2-6)	3 (2-5)	4 (2-6)	5 (3-6)	0.04*
**Number of study cycles,** ***n*** **(%)**					0.36
1	18 (17)	8 (22)	7 (19)	3 (8)	
2	13 (12)	6 (17)	4 (11)	3 8)	
3	18 (17)	6 (17)	4 (11)	8 (22)	
4	15 (14)	6 (17)	6 (17)	3 (8)	
5	8 (7)	3 (8)	3 (8)	2 (5)	
6	37 (34)	7 (19)	12 (33)	18 (49)	
**Fertility outcomes, n (%)**					
Positive pregnancy test	44 (40.4)	15 (41.7)	17 (47.2)	12 (32.4)	0.43
Ongoing pregnancy	33 (30.3)	13 (36.1)	12 (33.3)	8 (21.6)	0.36
Live birth	30 (27.5)	11 (30.6)	11 (30.6)	8 (21.6)	0.61
**DHD score, median(IQR)**					
DHD total (out of 130)	61 (50–72)	44 (40–49)	60 (57–63)	79 (72–87)	
Vegetables	4.8 (3.0–8.4)	2.6 (1.6–4.8)	4.7 (3.6–7.7)	8.4 (5.1–10)	<0.001*
Fruit	6.1 (2.6–10)	3.3 (2.0–6.4)	5.0 (2.3–9.1)	10 (6.1–10)	<0.001*
Grains	1.7 (0.0–3.9)	0.4 (0.0–1.6)	1.8 (0.4–3.8)	3.3 (1.0–5.1)	0.001*
Legumes	0.0 (0.0–0.0)	0.0 (0.0–0.0)	0.0 (0.0–0.0)	0.0 (0.0–0.0)	0.04*
Nuts	0.0 (0.0–4.3)	0.0 (0.0–0.0)	0.0 (0.0–0.8)	6.7 (0.0–10)	<0.001*
Dairy	6.2 (3.1–8.4)	4.0 (2.1–7.3)	6.5 (3.8–9.0)	6.4 (3.6–8.4)	0.07*
Fish	0.0 (0.0–2.7)	0.0 (0.0–0.3)	0.0 (0.0–2.7)	2.7 (0.0–10)	0.001*
Fats and oils	0.0 (0.0–4.3)	0.0 (0.0–0.0)	0 (0–0.3.3)	3.3 (0–10)	0.001*
Red meat	10 (10)	10 (10)	10 (10)	10 (10)	0.13*
Processed meat	2.0 (0.0–6.8)	1.8 (0.0–5.2)	0.9 (0.0–6.3)	4.2 (0.0–8.5)	0.35*
Sugar-sweetened beverages and fruit juices	4.0 (0–8.0)	0.0 (0.0–0.0)	5.0 (0.0–8.1)	6.7 (4.0–10)	<0.001*
Alcohol	10 (10)	10 (10)	10 (10)	10 (10)	1.00*
Tea	8.2 (1.7–10)	1.7 (0.0–9.4)	8.0 (3.4–10)	10 (7.8–10)	<0.001*

SD: standard deviation; IQR: inter quartile range; DHD: Dutch Healthy diet; BMI: body mass index; IVF: in-vitro fertilization.

Method, IVF (first known method) refers to the first method used in MNC-IVF as for some treatment method changes.

Food supplements (other than folic acid supplements) refer to supplements that were used besides folic acid supplements as all women used these.

DHD score: total can range between 0 and 130, each subcomponent ranges between 0 and 10.

* *p*-value from Kruskal–Wallis test.

# Missing data on supplemental intake *n* = 9, cause of infertility *n* = 1, duration of infertility *n* = 21, BMI *n* = 1.

### Cox proportional hazard analyses

Unadjusted analyses showed trends between a 10 points higher DHD2015-index and a lower frequency of a positive pregnancy test (HR = 0.88, 95%CI:0.74–1.04), ongoing pregnancy (HR = 0.80, 95%CI:0.64–1.00), and live birth (HR = 0.81, 95%CI:0.64–1.03) (Table 3). After adjustment for age, BMI, and smoking, associations became stronger and statistically significant for ongoing pregnancy (HR = 0.77, 95%CI: 0.62–0.96) and live birth (HR = 0.78, 95%CI: 0.62–0.98). Finally, additional analyses investigating the potential association between BMI, energy intake and age, and reproductive outcomes indicated an inverse association between a one unit higher BMI and all pregnancy outcomes (e.g. live birth: HR = 0.87, 95%CI: 0.77–0.98) while no associations were observed between daily energy intake, age and any of the pregnancy outcomes (Table 3 **)**.

**Table 3.** Hazard ratios of DHD2015-index, BMI and energy intake on IVF success

	DHD	BMI	Energy intake	Age
*N* = 109	Per 10 points	Per 1 kg/m^2^	Per 100 kcal/d	Per 1 year
Positive pregnancy test				
*N* (%) positive test	44 (40.4)			
Crude	0.88 (0.74–1.04)	0.90 (0.82–0.97)	1.01 (0.94–1.08)	1.00 (0.92–1.08)
Adjusted*	0.85 (0.71–1.01)	0.89 (0.82–0.98)	0.98 (0.91–1.06)	1.01 (0.93–1.10)
Ongoing pregnancy				
*N* (%) ongoing pregnancy	33 (30.3)			
Crude	0.80 (0.64–1.00)	0.90 (0.81–0.99)	1.04 (0.97–1.13)	1.01 (0.93–1.10)
Adjusted*	0.77 (0.62–0.96)	0.90 (0.81–1.00)	1.01 (0.93–1.10)	1.02 (0.94–1.11)
Live birth				
*N* (%) live birth	30 (27.5)			
Crude	0.81 (0.64–1.03)	0.87 (0.78–0.97)	1.03 (0.96–1.12)	1.01 (0.93–1.11)
Adjusted*	0.78 (0.62–0.98)	0.87 (0.77–0.98)	0.99 (0.91–1.08)	1.03 (0.94–1.12)

* Adjusted for BMI, age and smoking (not adjusted for when it is the exposure variable of interest).

### Stratified analyses

No significant interactions were observed between DHD scores and age (p = 0.61), BMI (p = 0.11), energy intake (p = 0.14), subfertility duration (p = 0.06), and method of fertility treatment (p = 0.06). Stratified analyses showed inverse associations between diet quality and all fertility treatment outcomes examined. To illustrate, among 87 couples undergoing ICSI treatment, the HR for a positive pregnancy test was 0.77 (95%CI: 0.63–0.95) (Table 4). However, in couples receiving IVF treatment (*n* = 22) a non-significant beneficial association was observed for a positive pregnancy test (HR = 1.12, 95%CI: 0.80–1.55) and no associations were observed for ongoing pregnancy and live birth (HR = 1.01, 95%CI: 0.58–1.74). Moreover, diet quality was inversely associated with all three pregnancy outcomes among women having a short duration of infertility (<34 months) (*n* = 44) (e.g. ongoing pregnancy; HR = 0.62, 95%CI: 0.43–0.91) while no associations were observed among women with a long duration of infertility (≥34 months) (*n* = 44). An inverse association between DHD2015-index and all pregnancy outcomes was observed among women with overweight (≥25 kg/m^2^) (*n* = 31) (e.g. ongoing pregnancy; HR = 0.61, 95%CI:0.39–0.94) while non-significant inverse associations were observed among women with normal weight (<25 kg/m^2^) (*n* = 77) (e.g. ongoing pregnancy; HR = 0.82, 95%CI: 0.64–1.06). Among women with high energy intakes (≥1854 kcal/day) (i.e. positive pregnancy test: HR = 0.76, 95%CI:0.59–0.98) a stronger inverse association was observed compared to women with low energy intakes (<1854 kcal/day) (i.e. positive pregnancy test; HR = 0.96, 95%CI:0.72–1.29).

**Table 4.** Hazard ratios of DHD-index on success of IVF stratified on method of treatment, duration of infertility, BMI, and energy intake

	Method of fertility treatment		Duration of infertility ^#^		BMI ^$^		Energy intake	
	ICSI	IVF	Short (<34 months)	Long (≥34 months)	Normal weight (<25 kg/m^2^)	Overweight (≥25 kg/m^2^)	Low (<1854 kcal/day)	High (≥1854 kcal/day)
*N*	87	22	44	44	77	31	54	55
DHD, median [IQR]	59 (50–71)	64 (48–75)	59 (51–72)	60 (50–68)	61 (50–75)	59 (47–66)	59 (48–67)	61 (51–79)
**Positive pregnancy test**								
*N* (%) positive test	34 (39.1)	10 (45.5)	19 (43.2)	19 (43.2)	34 (44.2)	10 (32.3)	23 (42.6)	21 (38.2)
Crude	0.83 (0.69–1.01)	1.07 (0.75–1.51)	0.72 (0.54–0.96)	1.02 (0.78–1.33)	0.92 (0.76–1.11)	0.68 (0.50–0.93)	0.97 (0.76–1.23)	0.81 (0.62–1.06)
Adjusted^1^	0.77 (0.63-0.95)	1.12 (0.80–1.55)	0.70 (0.51–0.97)	0.94 (0.69–1.28)	0.88 (0.72–1.09)	0.79 (0.58–1.06)	0.96 (0.72–1.29)	0.76 (0.59–0.98)
**Ongoing pregnancy**								
*N* (%) ongoing pregnancy	26 (29.9)	7 (31.8)	16 (36.4)	13 (29.5)	25 (32.5)	8 (25.8)	16 (29.6)	17 (30.9)
Crude	0.77 (0.58–1.01)	0.99 (0.63–1.54)	0.72 (0.53–0.97)	0.99 (0.70–1.40)	0.85 (0.66–1.09)	0.58 (0.40–0.85)	0.81 (0.62–1.05)	0.79 (0.57–1.10)
Adjusted^1^	0.71 (0.55–0.92)	1.01 (0.58–1.74)	0.62 (0.43–0.91)	0.86 (0.53–1.40)	0.82 (0.64–1.06)	0.61 (0.39–0.94)	0.80 (0.61–1.05)	0.73 (0.54–1.01)
**Live birth**								
*N* (%) live birth	23 (26.4)	7 (31.8)	13 (29.5)	13 (29.5)	24 (31.2)	6 (19.4)	16 (29.6)	14 (25.5)
Crude	0.76 (0.59-0.98)	0.99 (0.63-1.54)	0.75 (0.53–1.06)	0.99 (0.70–1.40)	0.86 (0.67–1.11)	0.54 (0.33–0.89)	0.81 (0.62–1.05)	0.83 (0.57–1.21)
Adjusted^1^	0.70 (0.55–0.90)	1.01 (0.58–1.74)	0.66 (0.44–0.99)	0.86 (0.53–1.40)	0.84 (0.65–1.08)	0.57 (0.32–0.99)	0.80 (0.61–1.05)	0.77 (0.54–1.08)

1 Adjusted for age, BMI and smoking status.

# Missing data on 21 women.

$ Missing data on 1 woman.

*P*-for-interaction for method of fertility treatment (*p* = 0.06), duration of infertility (*p* = 0.06), BMI (*p* = 0.11), and energy intake (*p* = 0.14).

### Analyses per component of DHD2015-index

No strong associations were observed between separate diet quality components and pregnancy outcomes (Supplemental Table 3). However, in the total population, a higher score for fish intake was associated with a lower probability of a positive pregnancy test (HR = 0.89, 95%CI:0.80–0.98). A higher score for legume intake was associated with higher chance on a positive pregnancy test among women receiving IVF treatment (HR = 1.20, 95%CI: 1.04–1.40). Furthermore, a higher score for tea consumption was associated with a lower probability of ongoing pregnancy and live birth among women receiving IVF treatment (HR = 0.75, 95%CI:0.63–0.88), while a higher score for processed meat, indicating a lower consumption, was associated with higher chances on ongoing pregnancy and live birth (HR = 1.31, 95%CI:1.07–1.62).

## Discussion

This is the first study to analyse the association between diet quality and MNC-IVF outcomes using extensive dietary data to determine diet quality based on an evaluated index. In this study, higher diet quality – but not energy intake – was, unexpectedly, inversely associated with all examined pregnancy outcomes after controlling for age, BMI and smoking in women receiving MNC-IVF. Inverse associations were most pronounced among women who underwent ICSI (i.e. also the majority of the study population), with a shorter duration of infertility, being overweight, and having higher energy intakes. On the other hand, expected inverse associations were observed between BMI and pregnancy outcomes. When analysing the separate components of the DHD2015- index, high fish, tea, and processed meat consumption were inversely associated with one or more of the fertility outcomes, while a higher intake of legumes was associated with a higher probability of a positive pregnancy test among women who underwent IVF.

Our findings of an inverse association between high diet quality and pregnancy chances are in contrast with a recent systematic literature review of 7 cohort studies, indicating that better adherence to the MedDiet, the Dutch ‘preconception’ diet and the ‘profertility’ diet was beneficially associated with pregnancy or live birth.^(16)^ For example, a Dutch study among 199 women receiving IVF with hyperstimulation observed higher chances of ongoing pregnancy among women with higher Preconception Dietary Risk scores (OR = 1.65, 95%CI: 1.08–2.52) while adjusting for age, smoking, diet partner, BMI of couple and treatment indication.^(18)^ Another study among 357 US women undergoing IVF/ICSI with hyperstimulation showed a positive association between adherence to the ‘pro-fertility diet’ and the probability of live birth (OR = 1.53, 95%CI:1.26–1.85), but not between another index, the Fertility diet, and any clinical outcomes (i.e. implantation, pregnancy or live birth) while adjusting for caloric intake, age, BMI, smoking status and physical exercise.^(44)^ Furthermore, a study by Sun and colleagues observed a non-significantly lower percentage of clinical pregnancy rate among women with higher adherence to the Mediterranean diet compared to women with lower adherence, i.e. 42.6% vs 50.9% clinical pregnancies (p = 0.30).^(45)^ Varying study findings may be explained by differences in study approaches, e.g. considered confounding factors, studied endpoints (e.g. first IVF treatment or all IVF attempts), different IVF procedures, definitions of dietary patterns,^(16)^ time-window of dietary assessment and usually limited sample sizes. Although, our analyses were adjusted for age, BMI and smoking, residual confounding might still have affected the observed associations. Secondary analyses of our study showed a negative association between BMI and pregnancy outcomes which is in agreement with previous studies.^(46–48)^ While a negative association was also expected between energy intake and pregnancy outcomes, this was not observed in this study.

Stratified analyses of our data particularly showed inverse associations between diet quality and pregnancy chances in women receiving ICSI treatment, while no or some positive trends were observed in women receiving IVF. As ICSI is often used as the primary treatment for severe male infertility, which is usually detected relatively early once a couple enters the ART trajectory, modification effects of duration and cause of infertility are challenging to disentangle in this study. Couples receiving ICSI had a significantly shorter duration of infertility and women tended to have a lower diet quality score. This was also observed in the study by Gaskins and colleagues (38 (36%) vs 24 (26%), T1 vs T4).^(44)^ It may be hypothesised that the potential effect of diet on ART successes depends on the allocated treatment method, which is associated to cause and likely also the duration of infertility. More specifically, being treated for severe male infertility, i.e. commonly treated via ICSI, may mask potential benefits from a healthy diet among the women undergoing the treatment but this does not explain the inverse associations observed. Finally, diet quality and pregnancy outcomes were inversely associated in the strata of women with higher energy intake and women being overweight. These unexpected results could potentially be explained by adaptation of the diet in the days before oocyte retrieval or group-specific misreporting.^(49)^

In contrast to dietary recommendations,^(50)^ separate analyses of the DHD-2015 components showed an inverse association between fish consumption and pregnancy chances. Despite being unexpected, this association might be due to elevated levels of contaminants in fish or the consumption of fried lean fish, which has lower omega fatty acid content and higher frying fat content.^(51)^ Inverse associations were also observed for tea consumption and pregnancy chances. Green tea and black teas, while generally considered healthy,^(52)^ contain caffeine, which should be limited before and during pregnancy.^(50)^ Limiting processed meat intake and increasing legume consumption appeared beneficial in this study, which is in line with the general dietary recommendations.^(50)^ These findings suggest that harmful components may have a stronger influence on pregnancy chances than healthy ones, but reversed causality as well as chance findings cannot be ruled out.

This study has limitations to consider. Firstly, the predominantly Caucasian sample restricts the generalizability of our findings to diverse ethnicities and dietary patterns. Second, while our study employed an IVF model that mirrors natural conception more closely than traditional IVF, it’s important to note that our findings from infertile couples undergoing ART cannot be generalised to naturally conceiving couples. Furthermore, the exclusion of participants lacking IVF or dietary data, as well as those with cycles involving the retrieval of more than one follicle, may have introduced potential selection bias. For instance, women with cycles resulting in multiple follicle retrievals may exhibit different physiological characteristics, such as varying hormonal responses. Additionally, while patients were broadly classified by tubal factor, male factor, or unexplained infertility, further diagnostic details were unavailable. The relatively small sample size may also raise concerns regarding statistical power, significance of results, and potential chance findings. To illustrate, our data suggest a positive association between higher diet quality and positive pregnancy tests in women undergoing IVF, but the small sample size in this subgroup calls for cautious interpretation. Another consideration is the use of a generalised diet quality index rather than a specialised preconception index. Nevertheless, we used a validated index for the Dutch general population^(26)^ to facilitate comparison between studies.^(16)^ Baseline data, such as BMI and smoking habits, were collected during the initial department visit. However, it’s crucial to acknowledge that the time intervals between this visit and the first MNC-IVF cycle differed among participants. This variation in timing might have impacted data reliability, particularly among those with longer time intervals between assessments. Furthermore, fluctuations in the number of food records among women may have resulted in less precise intake estimates, particularly for those with fewer records, due to increased measurement error stemming from their limited ability to capture day-to-day variations. While including women with fewer food records could dilute potential associations, excluding them would diminish statistical power and affect our study’s findings as well, even for food groups with limited day-to-day variation. Therefore, we included all participants with at least 2 food records in the analyses, as previous data has shown that 2 recording days can be acceptable for quantifying dietary intake of commonly consumed foods.^(53)^ Additionally, potentially limited variation among diet quality scores in our study could have masked any true associations between diet quality and pregnancy chances. Furthermore, relying on dietary intake data collected shortly before oocyte retrieval presents a limitation, as it may not accurately represent the habitual diet in the months preceding treatment. Dietary intake already affects the developing oocyte weeks prior to conception, while patients may have modified their diet specifically in the days leading up to treatment, potentially influenced by guidance from gynaecologists at UMCG directing patients to the Dutch Nutrition Centre webpage. In a separate analysis, we investigated the relationship between BMI, serving as a proxy for long-term dietary habits, and ART outcomes. Contrary to diet quality, we found that higher BMI was associated with lower pregnancy outcomes, as expected. Another hypothesis is that healthier eating habits developed in response to prolonged infertility, introducing the possibility of reversed causality.^(16)^ We also observed that women with a higher diet quality had a higher number of treatment cycles. However, the absence of differences in infertility duration across DHD2015-index tertiles and the absence of changes in diet over treatment cycles, as indicated by additional analyses (data not shown), suggests other potential explanations. Next to that, self-reported dietary data may have been influenced by systematic misreporting, such as socially desirable answers,^(54)^ as observed by lower reported energy and sugar-sweetened beverage intake among overweight women compared to those with normal weight (data not shown). To address potential misreporting, BMI adjustment was considered important in the analyses.^(49)^ Despite adjusting for several factors, interrelated covariates may have prevented complete adjustment, potentially obscuring the true association between diet quality and pregnancy outcomes. For instance, women undergoing IVF treatment tended to have longer infertility duration, higher BMI, older age, and slightly lower DHD scores. Despite these challenges, it is noteworthy that this study is the first to analyse the association between diet quality and MNC-IVF outcomes using extensive dietary data based on an evaluated index.^(26)^ Our participants closely mirrored the dietary patterns of Dutch women^(26,35^), and the use of MNC-IVF, with reduced hormonal stimulation enhances external validity compared to hyper stimulated IVF procedures. Moreover, the absence of frozen embryo use, eliminates the complexity of accounting for multiple preconception exposure periods associated with embryo retrieval and later transfer. Lastly, analyses examining the relationship between BMI and the outcomes support the internal validity of our study, despite some unexpected findings.

## Conclusion

Diet quality was inversely associated with MNC-IVF outcomes in the overall study population and specifically among those with ICSI treatment, short duration of infertility, overweight (BMI>=25 kg/m^2^) or high energy intake (>1854 kcal/day). Our findings also suggest that the association between diet quality and ART outcomes may depend on treatment type (ICSI or IVF), duration of infertility and potentially BMI and energy intake. When focusing on specific food groups, components to be limited may have the strongest dietary influences on pregnancy chances. To improve accuracy of collected dietary data, future studies should employ validated dietary assessment tools and if possible, dietary biomarkers to assess short-term and long-term dietary intake.^(55)^ Additionally, a larger sample size allowing for well-powered stratified analyses, and more power to adjust for additional potential confounders such as lifestyle factors of the male partner are warranted to better understand the association between diet and ART successes and to inform on the design of tailored nutritional intervention trials.

## Supporting information

Faessen et al. supplementary material Faessen et al. supplementary material

## Data Availability

Raw data may be obtained upon request via email to the corresponding author.

## Supplementary material

The supplementary material for this article can be found at https://doi.org/10.1017/jns.2024.97
