# Supplementary material for: Preconception diet quality and modified natural cycle in vitro fertilisation outcomes
Source: J Nutr Sci. 2025 Jan 23;14:e7. doi: 10.1017/jns.2024.97 (PMC11811871; doi:10.1017/jns.2024.97)
Supplement: Faessen et al. supplementary material [file S2048679024000971sup001.docx]

Supplemental table 1: Descriptive overview of nutrient, food group and diet quality data

| Dietary intake, mean +- SD |  | | | | |
| --- | --- | --- | --- | --- | --- |
|  | **Total** | **Low score (=<54)** | **Moderate score (54-66)** | **High score (=>66)** | **P-value** |
| Energy, kcal | 1924 (428) | 1976 (418) | 1914 (359) | 1912 (433) | 0.90 |
| Protein intake, % of EI  Plant protein, g/day  Animal protein, g/day | 16 (4)  6 (1)  9 (5) | 14 (3)  6 (1)  9 (3) | 15 (2)  6 (1)  9 (2) | 17 (4)  7 (1)  10 (5) | <0.01  <0.01  0.40 |
| Fat intake, % of EI  Saturated fats, g/day | 35 (7)  6 (1) | 31 (8)  5 (1) | 36 (5)  6 (1) | 36 (6)  6 (1) | <0.01  <0.01 |
| Carbohydrate intake, % of EI  Mono- and disaccharides, g/day  Polysaccharides, g/day | 47 (7)  23 (8)  24 (5) | 52 (10)  28 (11)  24 (5) | 47 (6)  22 (5)  25 (4) | 44 (6)  19 (4)  25 (5) | <0.01  <0.01  0.60 |
| Fiber intake, g/day | 19 (7) | 15 (4) | 19 (5) | 22 (7) | <0.01 |
| Water intake, mL/day | 2115 (638) | 1810 (582) | 2087 (518) | 2408 (637) | 0.01 |
| **Dietary intake DHD components, median (IQR)** |  |  |  |  |  |
| Vegetables, g/day | 95 (60-167) | 52 (33-96) | 94 (73-153) | 167 (101-203) | <0.01* |
| Fruit, g/day | 121 (53-213) | 66 (40-127) | 101 (46-182) | 214 (121-253) | <0.01* |
| Whole grains, g/day | 30 (0-64) | 7 (0-29) | 33 (7-63) | 53 (18-88) | <0.01* |
| Refined grains, g/day | 113 (70-159) | 128 (101-182) | 99 (78-148) | 91 (59-142) | 0.07* |
| Legumes, g/day | 0 (0-0) | 0 (0-0) | 0 (0-0) | 0 (0-0) | 0.06* |
| Nuts, g/day | 0 (0-6) | 0 (0-0) | 0 (0-1) | 10 (0-18) | <0.01* |
| Dairy cheese, g/day | 186 (94-259) | 119 (63-227) | 195 (112-272) | 193 (107-265) | 0.09* |
| Dairy, g/day | 150 (75-234) | 97 (57-201) | 166 (91-252) | 161 (100-225) | 0.10* |
| Cheese, g/day | 25 (11-40) | 20 (9-34) | 23 (12-31) | 32 (23-43) | 0.02* |
| Lean fish, g/day | 0 (0-0) | 0 (0-0) | 0 (0-7) | 0 (0-11) | 0.18* |
| Fatty fish, g/day | 0 (0-0) | 0 (0-0) | 0 (0-0) | 0 (0-26) | <0.01* |
| Liquid fat, g/day | 1 (0-8) | 0 (0-2) | 1 (0-8) | 6 (0-18) | <0.01* |
| Solid fat, g/day | 3 (0-8) | 3 (1-7) | 4 (2-12) | 1 (0-5) | 0.06* |
| Red meat, g/day | 3 (0-32) | 16 (0-41) | 5 (0-36) | 0 (0-20) | 0.08* |
| Processed meat, g/day | 40 (16-66) | 41 (24-63) | 46 (18-73) | 29 (7-62) | 0.23* |
| SSB & fruit juices, g/day | 150 (50-350) | 365 (249-528) | 125 (48-252) | 83 (0-150) | <0.01* |
| Alcohol, g/day | 0 (0-0)\| | 0 (0-0) | 0 (0-0) | 0 (0-0) | 0.91* |
| Tea, g/day | 370 (75-675) | 75 (0-425) | 360 (153-634) | 575 (350-938) | <0.01* |

P-value calculated from ANOVA test; *=p-value based on Kruskal Wallis test

Supplemental table 2: Descriptive overview stratified on ongoing pregnancy (no/yes)

|  | No pregnancy | Yes pregnancy | P-value |
| --- | --- | --- | --- |
| Participants (n) | n=76 | n=33 |  |
| **General characteristics** |  |  |  |
| Age, mean (SD) | 31.5 (3.6) | 31.4 (2.5) | 0.84 |
| BMI, mean (SD) | 23.9 (3.5) | 22.6 (3.2) | 0.07 |
| BMI => 25 | 23 (31) | 8 (24) | 0.65 |
| Age, mean (SD) | 32 (4) | 31 (3) | 0.84 |
| Current or past smoker before treatment, n(%) | 36 (48.0) | 9 (27.3) | 0.07 |
| Alcohol, n (%) | 43 (57.3) | 19 (59.4) | 1.00 |
| Drugs, n (%) | 1 (1.3) | 0 (0.0) | 1.00 |
| Food Supplement intake (other than folic acid) | 67 (94.4) | 29 (96.7) | 0.79 |
| Cause of infertility, n (%)  Male factor  Tubal (female)  Unexplained | 51 (68.0)  9 (12.0)  15 (20.0) | 24 (72.7)  6 (18.2)  3 (9.1) | 0.31 |
| Method, IVF (first known method), n (%) | 15 (19.7) | 7 (21.2) | 1.00 |
| Duration subfertility, months mean (SD) | 39 (24) | 36 (26) | 0.59 |
| **DHD score, median (IQR)** |  |  |  |
| DHD total (out of 130) | 62 (50-75) | 57 (47-66) | 0.13 |
| Vegetables | 4.9 (2.8-8.8) | 4.5 (3.1-7.4) | 0.67 |
| Fruit | 6.7 (3.0-10) | 4.7 (2.6-7.4) | 0.16 |
| Grains | 1.5 (0.0-3.9) | 1.7 (0.0-3.1) | 0.92 |
| Legumes | 0.00 (0.0-0.0) | 0.0 (0.0-0.0) | 0.45 |
| Nuts | 0.0 (0.0-6.0) | 0 (0-3.3) | 0.46 |
| Dairy | 6.4 (3.4-8.4) | 5.3 (3.0-6.9) | 0.22 |
| Fish | 0.0 (0.0-2.7) | 0.0 (0.0-2.7) | 0.17 |
| Fats and oils | 0.0 (0.0-4.5) | 0.0 (0.0-3.4) | 0.85 |
| Red meat | 10 (10-10) | 10 (10-10) | 0.79 |
| Processed meat | 1.3 (0.0-6.1) | 2.5 (0.0-8.4) | 0.29 |
| Sugar sweetened beverages and fruit juices | 5.3 (0.0-8.0) | 0.1 (0.0-5.5) | 0.07 |
| Alcohol | 10 (10-10) | 10 (10-10) | 0.25 |
| Tea | 8.3 (1.7-10) | 4.9 (1.4-10) | 0.72 |
| **Dietary intake, mean +- SD** |  |  |  |
| Energy, kcal | 1877 (418) | 1955 (359) | 0.35 |
| Protein intake, % of EI  Plant protein, g/day  Animal protein, g/day | 15 (4)  6 (1)  9 (4) | 15 (3)  6 (1)  9 (3) | 0.97  0.90  0.99 |
| Fat intake, % of EI  Saturated fats, g/day | 35 (5)  6 (1) | 34 (6)  6 (1) | 0.30  0.93 |
| Carbohydrate intake, % of EI  Mono- and disaccharides, g/day  Polysaccharides, g/day | 47 (8)  23 (9)  24 (5) | 49 (7)  23 (7)  25 (5) | 0.39  0.90  0.21 |
| Fiber intake, g/day | 19 (7) | 19 (4) | 0.93 |
| Water intake, mL/day | 2144 (671) | 2013 (507) | 0.32 |
| Vegetables, g/day | 98 (56-176) | 90 (63-147) | 0.65 |
| Fruit, g/day | 134 (59-220) | 94 (53-149) | 0.16 |
| Whole grains, g/day | 26 (0-66) | 31 (0-53) | 0.71 |
| Refined grains, g/day | 112 (67-137) | 125 (83-200) | 0.04 |
| Legumes, g/day | 0 (0-0) | 0 (0-0) | 0.51 |
| Nuts, g/day | 0 (0-9) | 0 (0-5) | 0.49 |
| Dairy cheese, g/day | 195 (103-267) | 160 (90-207) | 0.17 |
| Dairy, g/day | 162 (82-250) | 138 (61-196) | 0.14 |
| Cheese, g/day | 24 (10-36) | 26 (15-40) | 0.44 |
| Lean fish, g/day | 0 (0-11) | 0 (0-0) | 0.15 |
| Fatty fish, g/day | 0 (0-0) | 0 (0-0) | 0.42 |
| Liquid fat, g/day | 2 (0-7) | 0 (0-8) | 0.48 |
| Solid fat, g/day | 3 (0-9) | 3 (1-5) | 0.98 |
| Red meat, g/day | 1 (0-30) | 8 (0-35) | 0.59 |
| Processed meat, g/day | 43 (20-64) | 38 (8-68) | 0.43 |
| SSB & fruit juices, g/day | 119 (50-314) | 246 (113-467) | 0.03 |
| Alcohol, g/day | 0 (0-0) | 0 (0-0) | 0.24 |
| Tea, g/day | 375 (75-666) | 220 (63-719) | 0.84 |

P-value calculated from T-test. SSB=sugar sweetened beverages

Supplemental table 3: Association between scores per component and chance of IVF success

|  | Positive pregnancy test | | | Ongoing pregnancy | | | Live birth | | |
| --- | --- | --- | --- | --- | --- | --- | --- | --- | --- |
| HR (95%CI) | Total population | ICSI | IVF | Total population | ICSI | IVF | Total population | ICSI | IVF |
| n | 109 | 87 | 22 | 109 | 87 | 22 | 109 | 87 | 22 |
| n (%) success | 44 (40) | 34 (39) | 10 (46) | 33 (30) | 26 (30) | 7 (32) | 30 (28) | 23 (26) | 7 (32) |
| Fruit | 0.96 (0.88-1.04) | 0.98 (0.89-1.08) | 0.75 (0.52-1.10) | 0.97 (0.88-1.06) | 0.96 (0.87-1.07) | 0.95 (0.69-1.32) | 1.00 (0.90-1.10) | 1.00 (0.89-1.11) | 0.95 (0.69-1.32) |
| Vegetables | 1.06 (0.96-1.16) | 1.06 (0.94-1.19) | 1.01 (0.77-1.32) | 1.03 (0.92-1.16) | 0.99 (0.86-1.14) | 1.10 (0.86-1.42) | 1.05 (0.93-1.19) | 1.01 (0.86-1.19) | 1.10 (0.86-1.42) |
| Legumes | 0.98 (0.90-1.07) | 0.95 (0.85-1.06) | 1.20 (1.04-1.40) | 0.94 (0.84-1.05) | 0.92 (0.79-1.06) | 1.09 (0.92-1.28) | 0.94 (0.84-1.06) | 0.93 (0.79-1.08) | 1.09 (0.92-1.28) |
| Nuts | 0.95 (0.87-1.04) | 0.95 (0.87-1.05) | 0.96 (0.79-1.16) | 0.99 (0.90-1.09) | 0.96 (0.85-1.08) | 1.14 (0.98-1.33) | 1.01 (0.92-1.12) | 0.98 (0.87-1.11) | 1.14 (0.98-1.33) |
| Processed meat | 1.00 (0.93-1.08) | 0.97 (0.88-1.07) | 1.17 (0.98-1.40) | 1.05 (0.95-1.15) | 1.03 (0.92-1.15) | 1.31 (1.07-1.62) | 1.03 (0.93-1.14) | 1.00 (0.88-1.14) | 1.31 (1.07-1.62) |
| Red meat | 0.99 (0.88-1.12) | 0.99 (0.89-1.12) | 1.14 (0.87-1.51) | 0.96 (0.87-1.06) | 0.97 (0.86-1.08) | 1.38 (0.31-6.23) | 0.94 (0.85-1.04) | 0.95 (0.84-1.07) | 1.38 (0.31-6.23) |
| Fats & oils | 1.02 (0.96-1.09) | 1.02 (0.95-1.10) | 0.99 (0.84-1.16) | 1.00 (0.93-1.08) | 0.99 (0.90-1.09) | 0.95 (0.83-1.07) | 0.96 (0.88-1.05) | 0.95 (0.84-1.07) | 0.95 (0.83-1.07) |
| SSB + fruit juice | 1.01 (0.93-1.08) | 0.96 (0.88-1.05) | 1.15 (0.99-1.34) | 0.95 (0.87-1.04) | 0.93 (0.82-1.05) | 0.94 (0.75-1.19) | 0.96 (0.87-1.06) | 0.95 (0.83-1.08) | 0.94 (0.75-1.19) |
| Dairy | 0.98 (0.90-1.07) | 0.99 (0.90-1.09) | 0.99 (0.84-1.16) | 0.95 (0.86-1.04) | 0.98 (0.87-1.09) | 0.89 (0.78-1.01) | 0.96 (0.86-1.06) | 0.98 (0.87-1.11) | 0.89 (0.78-1.01) |
| Alcohol | / | / | / | / | **/** | **/** | / | **/** | **/** |
| Grains | 0.97 (0.86-1.10) | 0.93 (0.81-1.08) | 1.09 (0.84-1.42) | 0.99 (0.86-1.14) | 0.93 (0.78-1.11) | 1.19 (0.89-1.60) | 0.99 (0.85-1.14) | 0.92 (0.76-1.12) | 1.19 (0.89-1.60) |
| Fish | 0.89 (0.80-0.98) | 0.87 (0.75-1.01) | 0.92 (0.78-1.08) | 0.93 (0.84-1.04) | 0.91 (0.79-1.06) | 1.00 (0.85-1.18) | 0.91 (0.81-1.03) | 0.87 (0.72-1.05) | 1.00 (0.85-1.18) |
| Tea | 0.98 (0.91-1.06) | 0.99 (0.91-1.08) | 0.88 (0.72-1.08) | 0.93 (0.86-1.01) | 0.96 (0.88-1.04) | 0.75 (0.63-0.88) | 0.96 (0.87-1.05) | 0.99 (0.90-1.10) | 0.75 (0.63-0.88) |

A higher score per component means a healthier consumption of that component. For example; a higher score for processed meat actually means a lower consumption. A HR above 1; reflects a

beneficial association between a higher score for that component and fertility outcomes. Analyses were adjusted for BMI, age and smoking.
